# Induction of Dendritic Cell Maturation and Activation by a Potential Adjuvant, 2-Hydroxypropyl-β-Cyclodextrin

**DOI:** 10.3389/fimmu.2016.00435

**Published:** 2016-10-20

**Authors:** Sun Kyung Kim, Cheol-Heui Yun, Seung Hyun Han

**Affiliations:** ^1^Department of Oral Microbiology and Immunology, DRI, and BK21 Plus Program, School of Dentistry, Seoul National University, Seoul, South Korea; ^2^Department of Agricultural Biotechnology, Research Institute for Agriculture and Life Sciences, Seoul National University, Seoul, South Korea

**Keywords:** 2-hydroxypropyl-β-cyclodextrin, vaccine adjuvants, dendritic cells, maturation, lipid raft

## Abstract

2-Hydroxypropyl-β-cyclodextrin (HP-β-CD) is a chemically modified cyclic oligosaccharide produced from starch that is commonly used as an excipient. Although HP-β-CD has been suggested as a potential adjuvant for vaccines, its immunological properties and mechanism of action have yet to be characterized. In the present study, we investigated the maturation and activation of human dendritic cells (DCs) treated with HP-β-CD. We found that DCs stimulated with HP-β-CD exhibited a remarkable upregulation of costimulatory molecules, MHC proteins, and PD-L1/L2. In addition, the production of cytokines, such as TNF-α, IL-6, and IL-10, was modestly increased in DCs when treated with HP-β-CD. Furthermore, HP-β-CD-sensitized DCs markedly induced the proliferation and activation of autologous T lymphocytes. HP-β-CD also induced a lipid raft formation in DCs. In contrast, filipin, a lipid raft inhibitor, attenuated HP-β-CD-induced DC maturation, the cytokine expression, and the T lymphocyte-stimulating activities. To determine the *in vivo* relevance of the results, we investigated the adjuvanticity of HP-β-CD and the modulation of DCs in a mouse footpad immunization model. When mice were immunized with ovalbumin in the presence of HP-β-CD through a hind footpad, serum ovalbumin-specific antibodies were markedly elevated. Concomitantly, DC populations expressing CD11c and MHC class II were increased in the draining lymph nodes, and the expression of costimulatory molecules was upregulated. Collectively, our data suggest that HP-β-CD induces phenotypic and functional maturation of DCs mainly mediated through lipid raft formation, which might mediate the adjuvanticity of HP-β-CD.

## Introduction

Cyclodextrins are cyclic oligosaccharides composed of sugar molecules. These oligosaccharides consist of 6, 7, or 8 α-d-glucopyranose units bound *via* α-1,4-glycosidic linkages, namely, α-, β-, or γ-cyclodextrin, respectively. Cyclodextrins exhibit a bucket-shaped structure with a hydrophobic central cavity and a hydrophilic exterior (1). Cyclodextrins can efficiently form water-soluble inclusion complexes with hydrophobic molecules, which enhances the solubility and bioavailability of many insoluble compounds (2, 3). In addition, cyclodextrins improve and prolong the medicinal effects of drugs by controlling compound release, increasing their stability, and regulating the metabolism of the incorporated molecules (4). Due to these physicochemical properties, cyclodextrins are commonly utilized as excipients of pharmaceutical agents, food products, and cosmetics.

β-Cyclodextrin and some of its derivatives are widely used additives of commercial drugs because they are easy to produce, belong to generally recognized as safe (GRAS) materials for humans, and have improved solubility compared with the other cyclodextrins (4, 5). 2-Hydroxypropyl-β-cyclodextrin (HP-β-CD) is a chemically modified derivative of β-cyclodextrin that exhibits an enhanced safety profile compared with its naturally occurring parent compound (4). HP-β-CD is used as an excipient for cardiac dysrhythmia, inflammation, and fungal disease medications (6). Furthermore, HP-β-CD has been proposed as a vaccine adjuvant because it markedly enhances humoral immune responses to an influenza vaccine without any adverse effects (7). However, the immunological properties and action mechanism of HP-β-CD need to be further characterized for the human use.

Dendritic cells (DCs) are professional antigen-presenting cells that bridge the innate and adaptive immunities. Immature DCs are characterized by high endocytic activity coincident with a low expression of costimulatory molecules and cytokines (8). When immature DCs meet microbial antigens or damage-associated molecular patterns, they begin the process of maturation (8, 9). This process is accompanied by upregulation of (i) MHC associated with the antigen; (ii) costimulatory molecules including CD40, CD80, and CD86; and (iii) inflammatory cytokines such as IL-12, IL-6, and TNF-α (10). These phenotypic changes optimize conditions for T lymphocyte activation and differentiation (11, 12). Since mature DCs potently stimulate adaptive immunity better than immature DCs, many vaccine adjuvants currently under development are designed to efficiently induce functional maturation and activation of DCs (13–15). In the present study, we investigated immunological function of HP-β-CD by determining its ability to mature and activate DCs leading to the induction of adaptive immunity.

## Materials and Methods

### Reagents and Chemicals

2-Hydroxypropyl-β-cyclodextrin was purchased from Sigma-Aldrich (Saint Louis, MO, USA). Ficoll-Paque PLUS was obtained from GE Healthcare (Uppsala, Sweden). Fetal bovine serum (FBS) was purchased from GIBCO (Grand Island, NY, USA). RPMI-1640 medium and HyClone™ penicillin–streptomycin solution were from HyClone (Logan, UT, USA). Anti-human CD14 magnetic beads (clone: MΦP9) and anti-human CD3 magnetic beads (clone: HIT3a) were purchased from BD Biosciences (San Diego, CA, USA). Recombinant human granulocyte macrophage-colony stimulating factor (GM-CSF) and IL-4 were purchased from R&D Systems (Minneapolis, MN, USA) and CreaGene (Sungnam, Korea), respectively. Recombinant murine GM-CSF was obtained from CreaGene. 3,3′,5,5′-Tetramethylbenzidine (TMB) substrate and enzyme-linked immunosorbent assay (ELISA) kits for the quantification of human TNF-α, IL-6, IL-12p70, and IL-10, and mouse TNF-α and IL-6 were purchased from BioLegend (San Diego, CA, USA). 5,6-Carboxyfluorescein diacetate succinimidyl ester (CFDA-SE) and the Vybrant^®^ Alexa Fluor^®^ 594 lipid raft-labeling kit were obtained from Molecular Probes (Eugene, OR, USA). Filipin, bovine serum albumin (BSA), 2,2,2-tribromoethanol, 2-methyl-2-butanol, ovalbumin (OVA), and red blood cell (RBC)-lysis buffer were purchased from Sigma-Aldrich. Luria Bertani broth and Bacto™ Agar were purchased from BD Biosciences. FITC-labeled anti-human CD80 (clone: 2D10), PE-labeled anti-human CD83 (clone: HB15e), APC-labeled anti-human CD86 (clone: IT2.2), APC-labeled anti-PD-L1 (clone: 29E.2A3), PE-labeled anti-human PD-L2 (clone: 24F.10C12), APC-labeled anti-human CD25 (clone: BC96), PE-labeled anti-human CD4 (clone: RPA-T4), and PE-labeled anti-human CD8 (clone: RPA-T8) antibodies were purchased from BioLegend. FITC-labeled anti-human HLA-DR, DP, DQ (clone: Tu39) antibody for MHC class II, FITC-labeled anti-mouse CD86 (clone: GL-1) antibody, PE-labeled anti-mouse CD80 (clone: 16-10A1) antibody, PerCP-labeled anti-mouse CD11c (clone: N418) antibody, and FITC-labeled anti-mouse I-A^b^ (clone: 25-9-17) antibody for MHC class II were obtained from BD Biosciences. All isotype-matched antibodies were purchased from BD Biosciences or BioLegend. Horseradish peroxidase (HRP)-conjugated anti-mouse total IgG, anti-mouse IgG1, and anti-mouse IgG2a were purchased from Southern Biotech (Birmingham, AL, USA).

### Preparation of Human Monocyte-Derived DCs

Human peripheral blood samples donated by healthy adult male subjects (*n* = 15) were provided from the Korean Red Cross (Seoul, Korea) after obtaining informed consent. All experiments using human blood were conducted under the approval of the Institutional Review Board of Seoul National University. The peripheral blood was diluted in phosphate-buffered saline (PBS) and overlaid on the Ficoll-Paque PLUS, and peripheral blood mononuclear cells (PBMCs) were isolated by density-gradient centrifugation as previously described (16, 17). PBMCs were washed with PBS three times to remove platelets and the remaining Ficoll. To isolate CD14^+^ monocytes, PBMCs were incubated with anti-human CD14 magnetic beads for 30 min at room temperature. The cells were separated on a magnetic field, and then CD14^+^ cells were enriched by positive selection. CD14^+^ monocytes were suspended at a concentration of 2 × 10^6^ cells/ml in RPMI-1640 medium supplemented with 10% FBS and 1% penicillin–streptomycin solution. The isolated monocytes were differentiated into immature DCs in the presence of human recombinant GM-CSF (5 ng/ml) and IL-4 (10 ng/ml) for 6 days. Cell culture medium supplemented with GM-CSF and IL-4 was added every 3 days.

### Preparation of HKEC

*Escherichia coli* BL21 (DE3) strain obtained from Stratagene (La Jolla, CA, USA) was cultured in Luria Bertani broth at 37°C until reaching mid-log phase and then harvested by centrifugation. The harvested bacterial cells were resuspended in PBS and killed by heating at 60°C for 1 h. To confirm the complete killing, the heat-treated *E. coli* was plated onto Luria Bertani agar plates and cultured overnight at 37°C. No bacterial colonies were observed.

### Analysis of DC Phenotypes

Mouse BM-DCs (5 × 10^5^ cells/ml) were stimulated with either HP-β-CD (0, 0.1, or 1 mg/ml) or LPS (100 ng/ml) in the presence of murine GM-CSF (10 ng/ml) for 24 h. Human monocyte-derived DCs (2.5 × 10^5^ cells/ml) were stimulated with either HP-β-CD (0, 0.1, 0.3, or 1 mg/ml) or HKEC (1 × 10^7^ CFU/ml) in the presence of GM-CSF (2.5 ng/ml) and IL-4 (5 ng/ml) for 24 h. The unstimulated or stimulated DCs were then stained with fluorochrome-conjugated monoclonal antibodies specific to CD80, CD83, CD86, MHC class II, PD-L1, or PD-L2 for 30 min on ice, and then the cells were washed once with PBS. The geometric mean fluorescence intensity (MFI) of each group of DCs was obtained by flow cytometric analysis. More than 8,500 events were acquired for each group, and cell debris and dead cells were gated out. Phenotypes of DCs were analyzed using flow cytometry with FACSCalibur (BD Biosciences) and FlowJo software (TreeStar, San Carlos, CA, USA).

### Cytokine Quantification

The levels of cytokines produced by DCs were quantified by ELISA, as previously described (18). Briefly, mouse BM-DCs (5 × 10^5^ cells/ml) were stimulated with either HP-β-CD (0, 0.1, or 1 mg/ml) or LPS (100 ng/ml) in the presence of murine GM-CSF (10 ng/ml) for 24 h. Human monocyte-derived DCs (2.5 × 10^5^ cells/ml) were stimulated with either HP-β-CD (0, 0.1, 0.3, or 1 mg/ml) or HKEC (1 × 10^7^ CFU/ml) in the presence of GM-CSF (2.5 ng/ml) and IL-4 (5 ng/ml) for 24 h. The levels of TNF-α, IL-6, IL-12p70, and IL-10 in the culture supernatants were measured by ELISA kits according to the manufacturers’ instructions.

### Coculture of DCs with Autologous T Lymphocytes

To isolate CD3^+^ T lymphocytes, CD14^+^ monocyte-depleted PBMCs were incubated with anti-human CD3 magnetic beads for 30 min, and then CD3^+^ T lymphocytes were enriched by positive selection. The isolated CD3^+^ T lymphocytes were labeled with 10 μM CFDA-SE for 15 min at 37°C and washed with PBS. Immature DCs (2.5 × 10^5^ cells/ml) were stimulated with either HP-β-CD (0, 0.1, 0.3, or 1 mg/ml) or HKEC (1 × 10^7^ CFU/ml) in the presence of GM-CSF (2.5 ng/ml) and IL-4 (5 ng/ml) for 16 h. After removal of the culture supernatant, the DCs (5 × 10^4^ cells) were cocultured with carboxyfluorescein succinimidyl ester (CFSE)-labeled autologous CD3^+^ T lymphocytes (5 × 10^4^ cells) for 4–5 days. To analyze DC-mediated proliferation and activation of T lymphocyte subsets, the cells were stained with anti-human CD4, anti-human CD8, or anti-human CD25 antibodies and then analyzed by flow cytometry.

### Analysis of Lipid Raft Formation

Dendritic cells (2.5 × 10^5^ cells/ml) were stimulated with HP-β-CD (1 mg/ml) in the presence or absence of filipin (30 μg/ml) for 45 min. Cell staining was performed with the lipid raft-labeling kit according to the manufacturer’s instructions. Briefly, unstimulated or HP-β-CD-stimulated DCs were washed once with ice-cold PBS and stained with Alexa Fluor^®^ 594-conjugated CTB conjugate for 10 min on ice. The DCs were washed once with ice-cold PBS and incubated with rabbit serum containing anti-CTB antibodies for 10 min on ice to crosslink lipid rafts on the surface of the DCs. Formation of lipid raft on the DCs was analyzed by confocal laser scanning microscopy (Carl Zeiss MicroImaging GmbH, Jena, Germany). Fluorescence intensity of DCs was analyzed by ZEN software, Lite Edition (Carl Zeiss, Oberkochen, Germany).

### Immunization with OVA Plus HP-β-CD in Mice

Seven-week-old male C57BL/6 mice were purchased from Orient Bio (Sungnam, Korea) and maintained in a specific pathogen-free animal facility. All experiments using animals were conducted under the approval of the Institutional Animal Care and Use Committee of Seoul National University. Care and treatment of the animals were carried out in accordance with the approved guidelines. Mice were anesthetized by intraperitoneal injection of Avertin (2,2,2-tribromoethanol and 2-methyl-2-butanol) and administered with 20 μg OVA with or without 3 mg HP-β-CD through a hind footpad. The mice were maintained for 24 h or 7 days and sacrificed to obtain the draining lymph nodes and the blood, respectively.

### Analysis of DC Activation in the Draining Lymph Nodes

Twenty-four hours after the immunization with OVA in the presence or absence of HP-β-CD, draining lymph nodes, including popliteal and inguinal lymph nodes, were harvested and dissociated into a single cell suspension on a cell strainer (BD Biosciences). To analyze DC populations in the lymph nodes, the cells were stained with fluorochrome-conjugated antibodies specific to CD11c, MHC class II, CD86, and CD80 at 4°C for 30 min. CD11c^+^ MHC class II^+^ cells in the lymph nodes and their phenotypic changes upon the administration of OVA in the presence or absence of HP-β-CD were analyzed by flow cytometry using FACSVerse (BD Biosciences).

### Preparation of Mouse Bone Marrow-Derived DCs

Bone marrow-derived DCs (BM-DCs) were generated as previously described (19), with minor modifications. Briefly, BM cells were isolated from mouse femurs and tibias, and the RBCs were lysed with the RBC lysis buffer. Isolated BM cells were suspended in RPMI-1640 medium supplemented with 10% FBS and 1% penicillin–streptomycin solution and plated in petri dishes at 4 × 10^5^ cells/ml. The BM cells were differentiated into immature DCs in the presence of murine recombinant GM-CSF (20 ng/ml) for 7 days. Cell culture medium supplemented with GM-CSF was added every 4 days.

### Statistical Analysis

The statistical significance of differences between the experimental groups and the control group was analyzed using Student’s *t*-test. *P*-values less than 0.05 were considered statistically significant. Results are presented as mean value ± SD or SEM.

## Results

### HP-β-CD Induces Maturation of Human Monocyte-Derived DCs

**Figure 1:**
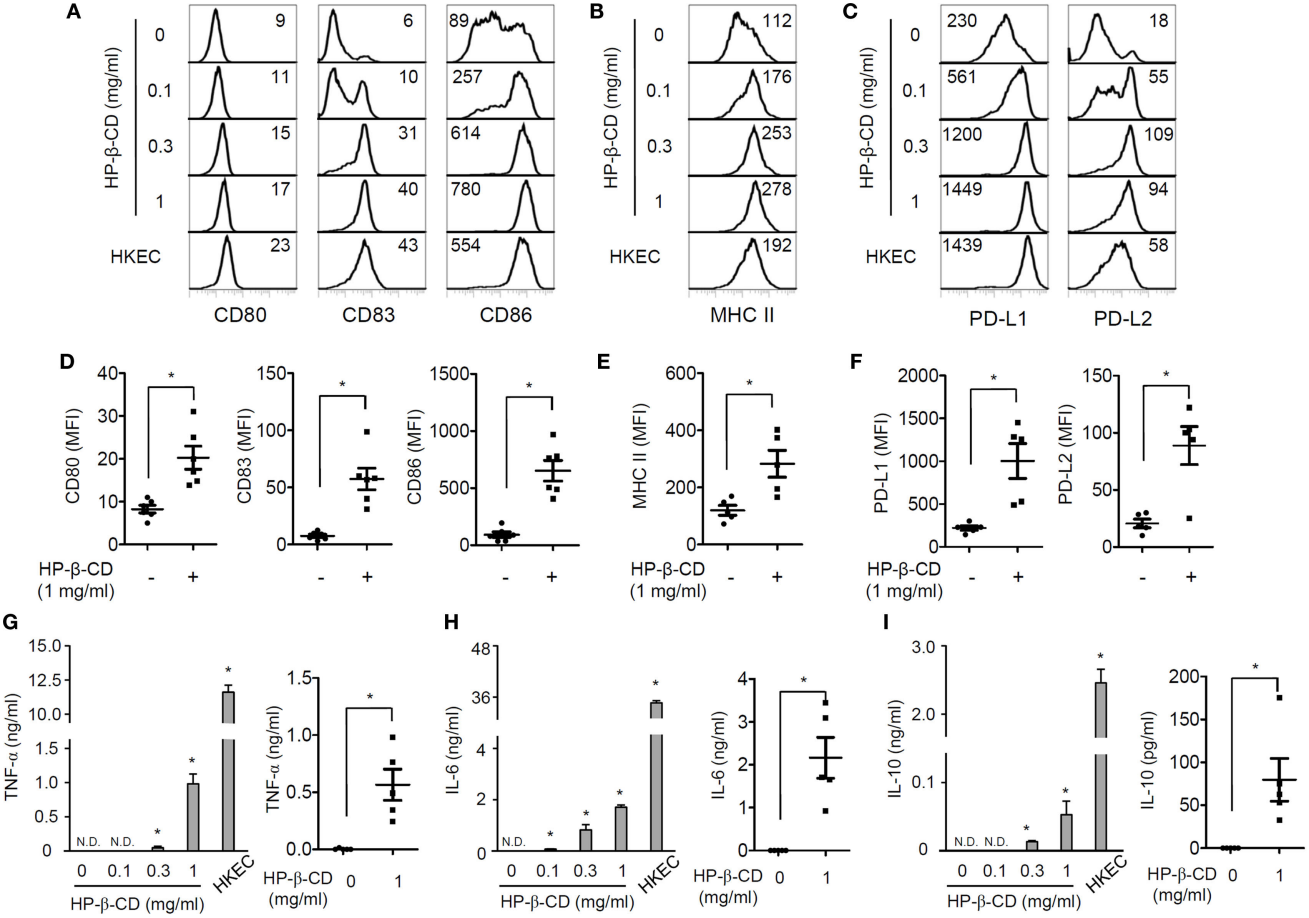
**HP-β-CD induces maturation of human monocyte-derived DCs**. Human CD14^+^ monocytes (2 × 10^6^ cells/ml) were differentiated into immature DCs in the presence of GM-CSF and IL-4 for 6 days. The monocyte-derived DCs (2.5 × 10^5^ cells/ml) were stimulated with HP-β-CD (0, 0.1, 0.3, or 1 mg/ml) in the presence of GM-CSF and IL-4 for 24 h. **(A–F)** Expression of **(A,D)** CD80, CD83, and CD86, **(B,E)** MHC class II, and **(C,F)** PD-L1 and PD-L2 was analyzed by flow cytometry. The number on each histogram indicates MFI of the DCs. **(D–F)** The scatter plots below the histograms indicate the average MFI of DCs ± SEM for each molecule (*n* = 5). The levels of **(G)** TNF-α, **(H)** IL-6, and **(I)** IL-10 in the DC culture supernatants were measured by ELISA. The scatter plots on the right side of each bar graph indicate the mean concentrations ± SEM of the cytokines (*n* = 5). HKEC (1 × 10^7^ CFU/ml) was used as a positive control. Statistical differences between compared groups were analyzed by paired Student’s *t*-test. N.D., not detected; *P < 0.05.

Maturation of DCs is an essential process for activating antigen-specific adaptive immunity and includes the upregulation of costimulatory molecules, MHC class I/II, and certain cytokines (12). Thus, we first examined the effects of HP-β-CD on the phenotypic maturation and cytokine production in human monocyte-derived DCs. Notably, HP-β-CD was not cytotoxic to DCs at concentrations up to 1 mg/ml (Figure S1A in Supplementary Material). Stimulation with HP-β-CD remarkably augmented the expression of costimulatory molecules, such as CD80, CD83, and CD86 (Figures 1A,D), together with MHC class II (Figures 1B,E). Additionally, HP-β-CD-treated DCs exhibited increased expression of inhibitory molecules, such as PD-L1 and PD-L2 (Figures 1C,F). Moreover, HP-β-CD weakly increased the expression of proinflammatory cytokines, including TNF-α and IL-6, in a dose-dependent manner (Figures 1G,H). Furthermore, HP-β-CD treatment slightly increased IL-10 expression in DCs (Figure 1I), and IL-12p70 was not detected in the culture supernatant of HP-β-CD-treated DCs (data not shown). These results suggest that HP-β-CD upregulates the expression of maturation markers coincident with weak induction of cytokines in human monocyte-derived DCs.

### HP-β-CD-Sensitized DCs Elicit Autologous T Lymphocyte Proliferation and Activation

**Figure 2:**
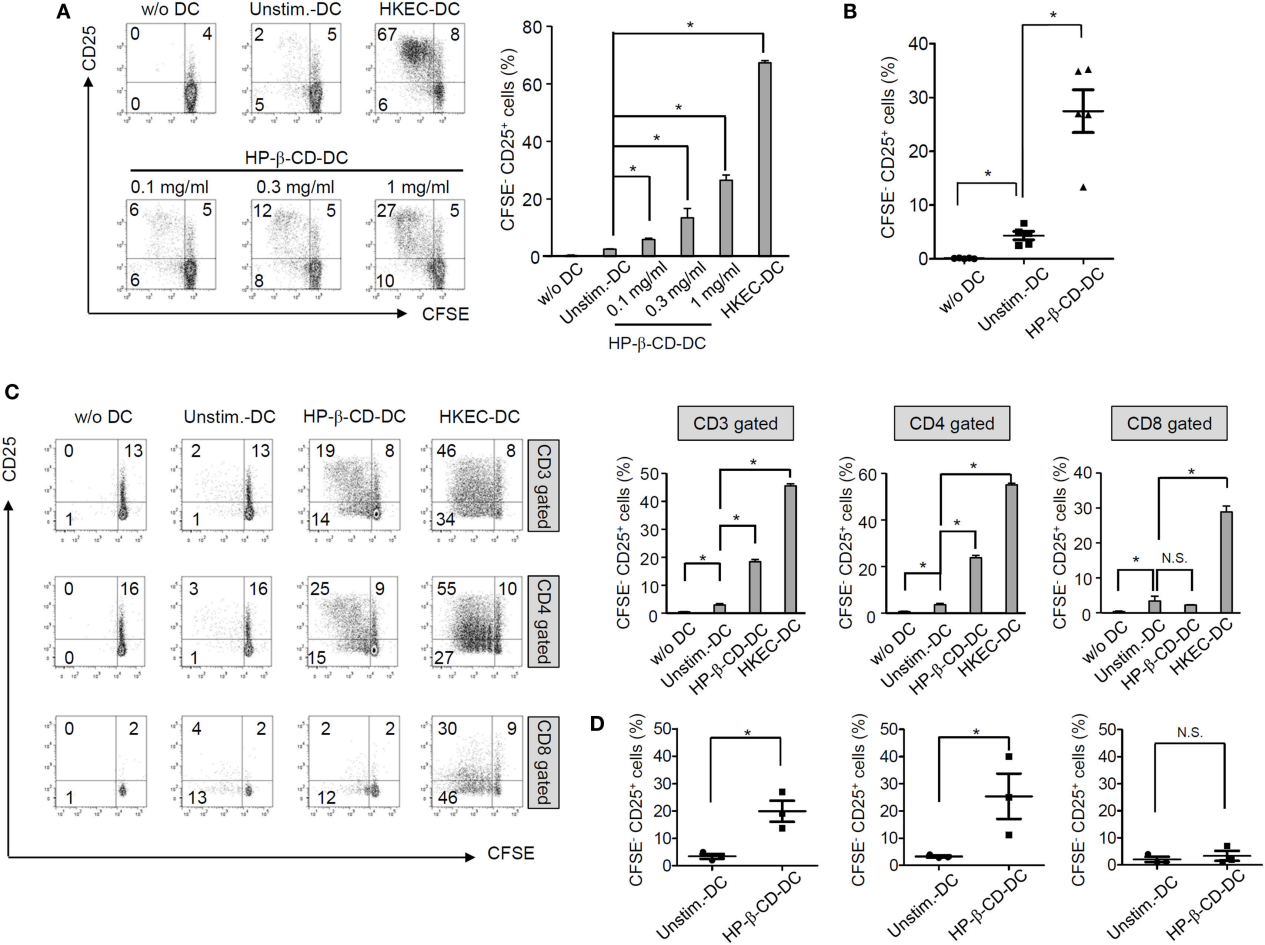
**HP-β-CD-sensitized DCs elicit the proliferation and activation of autologous T lymphocytes**. Human monocyte-derived DCs (2.5 × 10^5^ cells/ml) were stimulated with HP-β-CD (0, 0.1, 0.3, or 1 mg/ml) for 16 h. Then, the cells (5 × 10^4^ cells) were cocultured with CFSE-labeled autologous CD3^+^ T cells (5 × 10^4^ cells) for 4 days. **(A)** T lymphocyte proliferation and CD25 expression were analyzed by flow cytometry. The bar graph beside the histograms displays the average of triplicate measurements for the proliferation of CD25^+^ T lymphocytes. **(B)** A scatter plot indicates activated T lymphocytes by DCs (*n* = 5). **(C)** The proliferation and CD25 expression levels of CD3^+^, CD4^+^, or CD8^+^ T lymphocytes were analyzed by flow cytometry. The bar graphs on the right side of the histograms exhibit the average of triplicate measurements for the proliferation of CD25^+^ T lymphocytes. **(D)** Scatter plots exhibit activated CD3^+^, CD4^+^, or CD8^+^ T lymphocytes by DCs (*n* = 3). The immune responses of T lymphocytes cultured with HKEC-stimulated DCs are shown as positive controls. The numbers on the histograms indicate the percentages of T lymphocytes in each quadrant. *P < 0.05. Unstim.-DC indicates T lymphocytes cocultured with unstimulated DCs.

To examine whether HP-β-CD potentiates the T lymphocyte-activating ability of DCs, unstimulated or HP-β-CD-stimulated DCs were cocultured with autologous T lymphocytes, and the extents of T lymphocyte proliferation and activation were analyzed. HP-β-CD-sensitized DCs significantly induced T lymphocyte proliferation and CD25 expression, an activation marker of T lymphocytes (Figures 2A,B). The enhancement of proliferation and CD25 expression was observed in CD4^+^ T lymphocytes but not in CD8^+^ T lymphocytes (Figures 2C,D). However, HP-β-CD did not directly enhance the proliferative activity or CD25 expression of the T lymphocytes, indicating that HP-β-CD requires the help of DCs to activate T lymphocytes (Figures S2A,B in Supplementary Material). Therefore, the results suggest that HP-β-CD potentiates the ability of DCs to induce CD4^+^ cells.

### HP-β-CD Triggers Lipid Raft Formation on the DC Plasma Membrane

**Figure 3:**
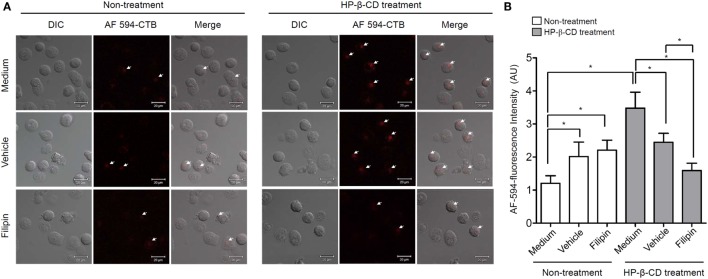
**HP-β-CD triggers lipid raft formation in DCs**. Human monocyte-derived DCs (2.5 × 10^5^ cells/ml) were stimulated with HP-β-CD (1 mg/ml) in the presence or absence of filipin (30 μg/ml) or methanol as a vehicle control for 45 min. **(A)** Stimulated DCs were stained with Alexa Fluor^®^ 594-conjugated CTB, and the formation of lipid rafts on the cell surface was examined by confocal microscopy. **(B)** Fluorescence intensity of DCs was analyzed by ZEN software. DIC, differential interference contrast; AU, arbitrary unit. Images shown are representative of three similar results.

Lipid rafts are hydrophobic microstructures that play an important role as signal transduction platforms in many eukaryotic cells (20). Since HP-β-CD interacts with cellular cholesterol (21), an essential component of lipid rafts, we examined whether HP-β-CD induces lipid raft formation on the plasma membrane based on the hypothesis that lipid rafts are involved in the HP-β-CD-induced DC maturation. DCs were stimulated with HP-β-CD in the presence or absence of filipin, which disrupts a lipid raft formation, and fluorochrome-conjugated CTB was used to detect the formation of lipid rafts. As shown in Figure 3, HP-β-CD triggered lipid raft formation on the surface of DCs, whereas such effect was blocked by preexposure to the lipid raft inhibitor filipin.

### Inhibition of Lipid Raft Formation Reduces HP-β-CD-Induced DC Maturation

**Figure 4:**
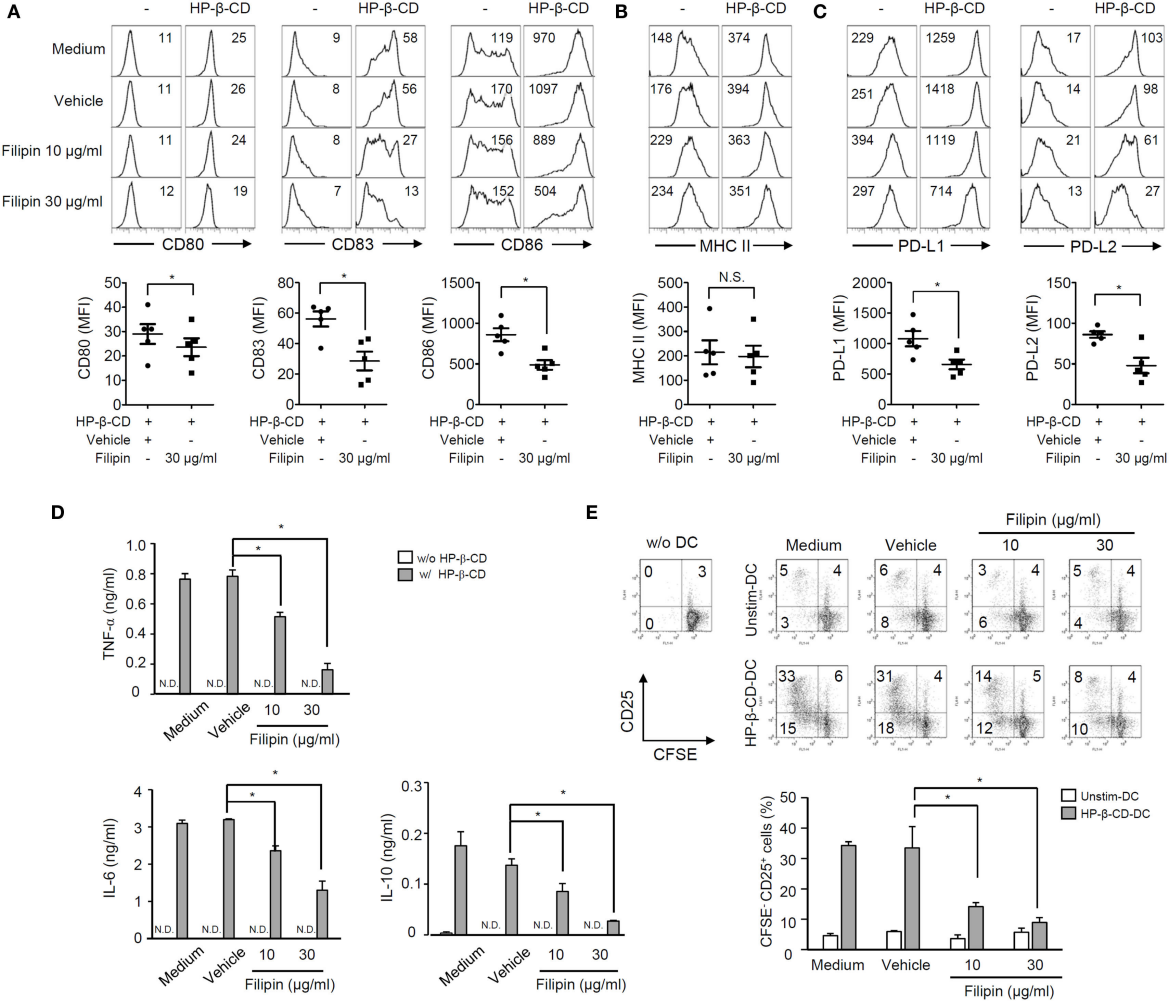
**Inhibition of lipid raft formation attenuates DC maturation, cytokine production, and autologous T cell activation induced by HP-β-CD**. Human monocyte-derived DCs (2.5 × 10^5^ cells/ml) were pretreated with filipin (10 and 30 μg/ml) or methanol as a vehicle control for 1 h, followed by stimulation with HP-β-CD (1 mg/ml) for another 24 h. **(A–C)** The expression of costimulatory molecules, MHC class II, and PD-L1/L2 was analyzed by flow cytometry. The numbers on the histograms indicate the MFIs of the cells. Scatter plots under the histogram show MFI of DCs (*n* = 5). Statistical differences between compared groups were analyzed by paired Student’s *t*-test. N.S., not significant; *P < 0.05. **(D)** The concentrations of TNF-α, IL-6, and IL-10 in the supernatants were measured by ELISA. **(E)** Unstimulated or HP-β-CD-stimulated DCs (5 × 10^4^ cells) were cocultured with CFSE-labeled autologous T lymphocytes (5 × 10^4^ cells) for 5 days. The levels of proliferation and CD25 expression of the T lymphocytes were analyzed by flow cytometry. The numbers on the histograms indicate the percentages of cells in each quadrant. The bar graph below the histograms displays the average of triplicate measurements ± SD for the frequencies of CD25^+^ T lymphocytes with proliferative ability. Unstim.-DC indicates T lymphocytes cocultured with unstimulated DCs. Results shown are representative of three independent experiments. Statistical differences between compared groups were analyzed by Student’s *t*-test. N.D., not detected; *P < 0.05.

Next, we further determined the role of lipid rafts in HP-β-CD-mediated DC maturation by blocking the lipid raft formation using filipin. Of note, treatment with filipin and/or HP-β-CD was not cytotoxic to DCs (Figure S1B in Supplementary Material). As shown in Figures 4A–C, treatment with filipin remarkably attenuated HP-β-CD-elicited expression of surface costimulatory molecules, PD-L1/L2, but not MHC class II. In addition, HP-β-CD-mediated induction of TNF-α, IL-6, and IL-10 in DCs was significantly reduced upon the inhibition of lipid raft (Figure 4D). Furthermore, lipid raft inhibition in DCs stimulated with HP-β-CD abrogated their ability to activate T lymphocytes (Figure 4E). These results suggest that HP-β-CD requires the formation of lipid rafts to trigger DC maturation and further to activate T lymphocytes.

### HP-β-CD Potentiates Humoral Immune Responses to Coadministered Antigens and DC Activation in the Draining Lymph Nodes in Mice

**Figure 5:**
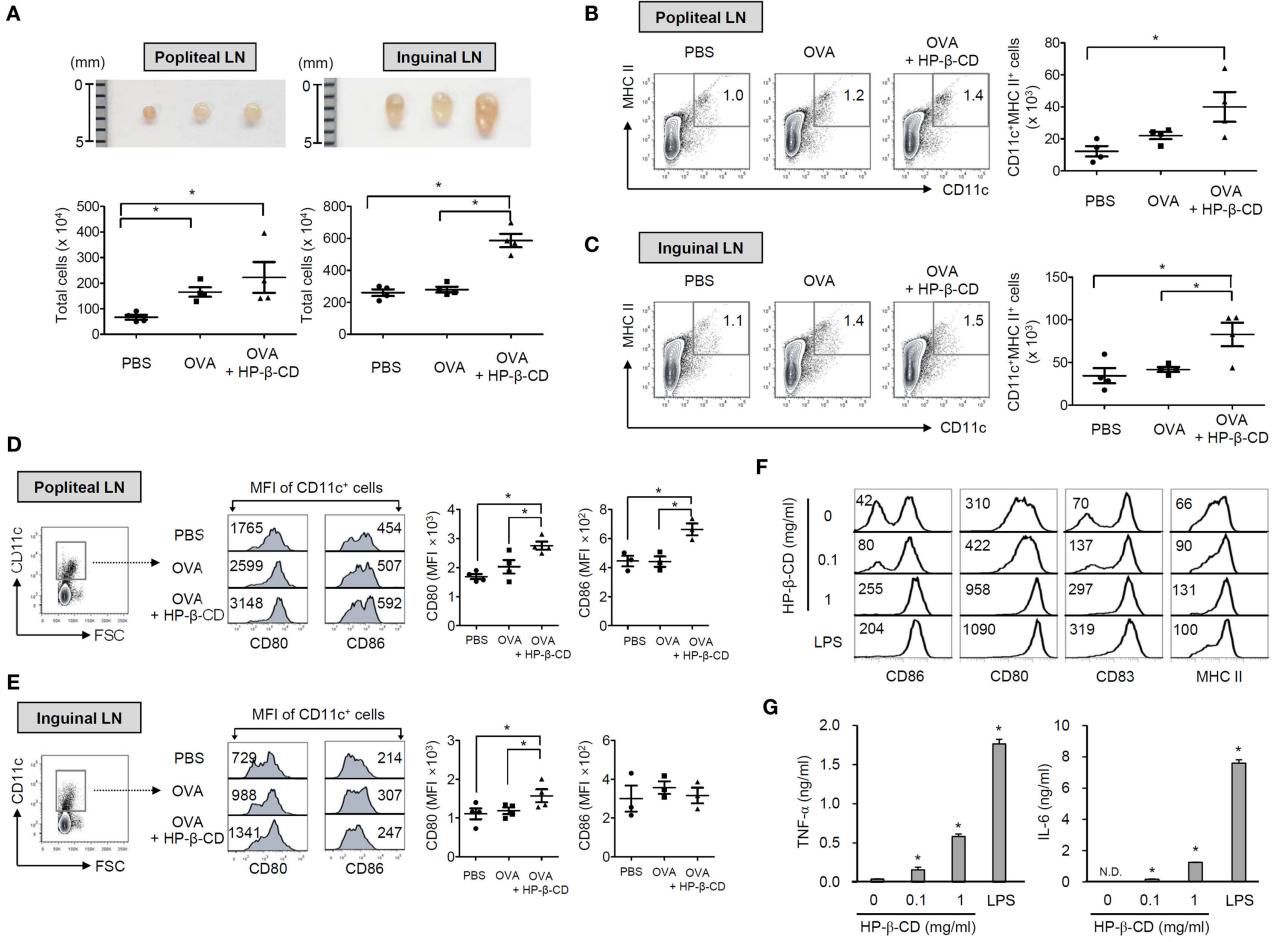
**HP-β-CD induces DC maturation and activation *in vivo* and *in vitro* mouse models**. C57BL/6 mice were immunized with 20 μg OVA in the presence or absence of 3 mg HP-β-CD through a hind footpad. Twenty-four hours after the immunization, DC populations were analyzed in the draining lymph nodes including popliteal and inguinal lymph nodes. **(A)** Size of the lymph nodes and the total cell numbers were measured (*n* = 4). CD11c^+^ MHC class II^+^ cells in the draining lymph nodes, **(B)** popliteal and **(C)** inguinal lymph nodes were analyzed by flow cytometry (*n* = 4). Expression of CD80 and CD86 on CD11c^+^ cells was analyzed in the draining lymph nodes, **(D)** popliteal and **(E)** inguinal lymph nodes by flow cytometry. Scatter plots on the right side the histograms indicate MFI of DCs for CD80 (*n* = 4) and CD86 (*n* = 3). **(F)** BM-DCs (5 × 10^5^ cells/ml) were stimulated with HP-β-CD (0.1 or 1 mg/ml) or LPS (100 ng/ml) in the presence of murine GM-CSF for 24 h. The surface expression of various maturation markers, including **(F)** CD80, CD83, CD86, and MHC class II molecules on the DCs, was analyzed by flow cytometry. **(G)** The amounts of TNF-α and IL-6 in the culture supernatants of DCs were quantified by ELISA. The number on each histogram indicates percentage or MFI of the DCs. LN, lymph node. N.D., not detected. N.S., not significant. *P < 0.05. The result shown is a representative of four similar experiments.

Next, we determined whether HP-β-CD has an adjuvanticity with a mouse footpad immunization model (22). To immunize mice, OVA with or without HP-β-CD was injected through a hind footpad, and titers of serum antibodies specific to OVA were determined. Coadministration with HP-β-CD and OVA efficiently increased OVA-specific total IgG in the blood (Figure S3A in Supplementary Material). IgG1 was the major antibody subtype induced following immunization (Figure S3B in Supplementary Material), and no IgG2a antibodies were detected (data not shown). As DCs are crucial in the mediation of naive T cell responses, we subsequently analyzed DC populations in the draining lymph nodes, including popliteal and inguinal lymph nodes upon administration of OVA with or without HP-β-CD. Mice administered with OVA with HP-β-CD showed an increase in the size and cell number of the draining lymph nodes (Figure 5A). OVA administration with HP-β-CD markedly augmented the number of CD11c^+^ MHC class II^+^ cells in the popliteal and inguinal lymph nodes (Figures 5B,C). In addition, the DCs showed an upregulation in the expression of costimulatory molecules, including CD80 and CD86 (Figures 5D,E). To determine whether DC activation in the draining lymph nodes of mice administered with OVA and HP-β-CD is directly attributed to stimulatory functions of HP-β-CD, we examined HP-β-CD effects on the maturation of BM-DCs generated *in vitro*. HP-β-CD markedly upregulated the expression of surface costimulatory molecules, such as CD80, CD83, and CD86, and MHC class II (Figure 5F). In addition, HP-β-CD-treated BM-DCs modestly increased the production of proinflammatory cytokines including TNF-α and IL-6 in BM-DCs compared to LPS-stimulated BM-DCs (Figure 5G). Collectively, these results suggest that HP-β-CD-induced DC maturation and activation *in vivo* that might be necessary for the adjuvanticity.

## Discussion

Dendritic cells play a pivotal role in the induction of antigen-specific adaptive immune response by presenting the antigens to T cells and activating appropriate subtypes of T cells. HP-β-CD has long been utilized as a solubilizer and a delivery compound of hydrophobic drugs due to its physicochemical properties (6). However, recent studies have reported a novel beneficial effect of HP-β-CD on the immunogenicity of vaccines (7, 23) as well as on the progression of incurable metabolic disorders (24) and viral infections (25). Although the previous findings have suggested that HP-β-CD could be a potential vaccine adjuvant (7, 23), the effects of HP-β-CD on vaccine immunogenicity and DC properties have been poorly investigated. Here, we demonstrated that HP-β-CD has an adjuvanticity to OVA, and the maturation of DCs found in the draining lymph nodes in a mouse footpad immunization model. *In vitro* studies showed that HP-β-CD induces the maturation of DCs to induce the proliferation and activation of autologous T lymphocytes. Mechanism studies further showed that the lipid raft formation in DCs is essential for the HP-β-CD-induced DC maturation and its subsequent activation of autologous T cells. These results suggest that HP-β-CD is a promising vaccine adjuvant to potently induce the maturation and activation of DCs.

In the present study, we demonstrated that HP-β-CD has an adjuvanticity. Administration of HP-β-CD efficiently augmented the antigen-specific IgG in the blood. Notably, HP-β-CD predominantly induced IgG1 but not IgG2, indicating preferential enhancement of Th2 responses rather than Th1 responses. Consistent with our findings, intranasal or subcutaneous administration of HP-β-CD increased the immunogenicity of influenza vaccines (7, 23). HP-β-CD seems to be appropriate as a mucosal adjuvant because it induces antigen-specific IgA and IgG in the airway mucosal tissues as well as in the blood (23).

Dendritic cell maturation is a prerequisite for the induction of adaptive immune response. Many previous studies have demonstrated that current vaccine adjuvants, such as aluminum salt (alum), cholera toxin (CT), and monophosphoryl lipid A (MPLA), can efficiently induce DC maturation (26–28). Here, we observed that HP-β-CD increased DCs in the draining lymph nodes as well as the upregulation of DC costimulatory molecules when coadministered with antigen. Additionally, HP-β-CD induced a marked increase in the expression of CD80, CD83, CD86, and MHC proteins on BM-DCs and human monocyte-derived DCs. Furthermore, stimulation with HP-β-CD resulted in weak but significant production of TNF-α, IL-6, and IL-10 in DCs. Therefore, like other adjuvants, HP-β-CD appears to be capable of stimulating DC maturation. However, in contrast to our observation, a previous study found that HP-β-CD did not induce a maturation marker of CD11c^+^ DCs in the draining lymph node, even though it potentiated DC antigen uptake (7). Given the differences in a route of injection and doses of HP-β-CD, functional mode of DC activation in the tissues might be different.

Here, we found that HP-β-CD-sensitized DCs markedly induced the proliferation and activation of T lymphocytes, especially CD4^+^ cells, implying that HP-β-CD can potentially enhance Th-dependent immune responses. Notably, a study of mice immunized with an influenza vaccine suggested that HP-β-CD is a competent adjuvant capable of eliciting follicular Th cells and antibody production (7). Furthermore, other cyclodextrins, such as sulfolipo-cyclodextrin and dimethyl-β-cyclodextrin, have been found to enhance antibody responses in a T lymphocyte-dependent manner (29, 30). Given the fact that enhanced T cell responses have previously been observed with many existing adjuvants, including MPLA, saponin, and CT (31, 32), this enhancement appeared to be mediated through DC activation. These adjuvants influence the types of adaptive immune responses that occur by modulating T lymphocyte differentiation. For instance, alum is an established potentiator of Th2-mediated humoral immunity (33). In addition, MPLA has been shown to preferentially induce Th1-skewed immune responses (34), whereas CT provokes Th1, Th17, and Th2 responses (35). Considering the previous finding that HP-β-CD administration markedly induces IL-13 and IL-5 production, HP-β-CD has been proposed to elicit Th2 responses (7).

In the present study, we observed that HP-β-CD-induced lipid raft formation and contributed to DC maturation. Specifically, filipin-mediated inhibition of lipid rafts abrogated HP-β-CD-mediated phenotypic changes and functional activation of DCs. In line with this observation, many previous studies have demonstrated lipid raft involvement in the activation of various immune cells. Dispersion of lipid rafts has been shown to impair CD1a-mediated antigen presentation by DCs and subsequent activation of T lymphocytes (36). Furthermore, alum adjuvanticity has been proposed to have a critical relationship with the formation of signaling platforms *via* lipid sorting on DCs (26).

2-Hydroxypropyl-β-cyclodextrin can interact with cellular cholesterol (21); however, the precise effects of HP-β-CD on cholesterol seem to depend on its concentration. Low doses of HP-β-CD (below 1 mM) induce efflux or intermembrane transport of cholesterol, while high concentrations (10–100 mM) deplete the lipid molecules from the cell membrane (37, 38). In the present study, DCs were treated with up to 1 mg/ml HP-β-CD, which is equivalent to 1.4 mM. This concentration is relatively low and is likely to mediate cholesterol accumulation and lipid raft formation on the cell membrane rather than to deplete cholesterol. In light of the observation that cholesterol accumulation in the membrane of human monocytes promotes the association of immuno-stimulatory receptor complexes, cholesterol loading in the plasma membrane is believed to be crucial for eliciting inflammatory immune responses (39). Since HP-β-CD can transport free cholesterol to the plasma membrane (40), HP-β-CD is likely to play a role in the formation of cholesterol-rich lipid rafts in DCs, thereby activating signals required for their phenotypic and functional maturation.

Recently, potential issues in relation to the efficacy and safety of vaccine adjuvant have been raised (41–44). Thus, demand has increased for new adjuvants with minimal adverse effects and enhanced capacity to stimulate antigen-specific adaptive immune responses. HP-β-CD is GRAS in many Asian and European countries and is already widely utilized in various commercial products, including food and drugs. Our findings potentially inform the future application and improvement of vaccine adjuvants with HP-β-CD.

## Author Contributions

SH conceived the idea. SH and SK designed the experiments. SK and SH performed the experiments and/or interpreted the data. C-HY provided critical comments. All authors contributed to discussion of the results followed by writing and reviewing the manuscript.

## Conflict of Interest Statement

The authors declare that the research was conducted in the absence of any commercial or financial relationships that could be construed as a potential conflict of interest.

## References

[1] Loftsson T Brewster ME. Pharmaceutical applications of cyclodextrins.1. Drug solubilization and stabilization. J Pharm Sci (1996) 85:1017–25. 10.1021/Js950534b 8897265

[2] Rawat S Jain SK. Solubility enhancement of celecoxib using beta-cyclodextrin inclusion complexes. Eur J Pharm Biopharm (2004) 57:263–7. 10.1016/j.ejpb.2003.10.020 15018983

[3] Jin X Zhang ZH Sun E Jia XB beta-cyclodextrin assistant flavonoid glycosides enzymatic hydrolysis. Pharmacogn Mag (2013) 9:11–8. 10.4103/0973-1296.117851 PMC3798134 24143039

[4] Kurkov SV Loftsson T. Cyclodextrins. Int J Pharm (2013) 453:167–80. 10.1016/j.ijpharm.2012.06.055 22771733

[5] Brewster ME Loftsson T. Cyclodextrins as pharmaceutical solubilizers. Adv Drug Deliv Rev (2007) 59:645–66. 10.1016/j.addr.2007.05.012 17601630

[6] Loftsson T Jarho P Masson M Jarvinen T Cyclodextrins in drug delivery. Expert Opin Drug Deliv (2005) 2:335–51. 10.1517/17425247.2.1.335 16296758

[7] Onishi M Ozasa K Kobiyama K Ohata K Kitano M Taniguchi K Hydroxypropyl-beta-cyclodextrin spikes local inflammation that induces Th2 cell and T follicular helper cell responses to the coadministered antigen. J Immunol (2015) 194:2673–82. 10.4049/jimmunol.1402027 25681338 PMC4470223

[8] Mellman I Steinman RM Dendritic cells: specialized and regulated antigen processing machines. Cell (2001) 106:255–8. 10.1016/S0092-8674(01)00449-4 11509172

[9] Fang H Ang B Xu X Huang X Wu Y Sun Y TLR4 is essential for dendritic cell activation and anti-tumor T-cell response enhancement by DAMPs released from chemically stressed cancer cells. Cell Mol Immunol (2014) 11:150–9. 10.1038/cmi.2013.59 24362470 PMC4003380

[10] Dudek AM Martin S Garg AD Agostinis P. Immature, semi-mature, and fully mature dendritic cells: toward a DC-cancer cells interface that augments anticancer immunity. Front Immunol (2013) 4:438. 10.3389/fimmu.2013.00438 24376443 PMC3858649

[11] Osorio F Fuentes C Lopez MN Salazar-Onfray F Gonzalez FE. Role of dendritic cells in the induction of lymphocyte tolerance. Front Immunol (2015) 6:535. 10.3389/fimmu.2015.00535 26539197 PMC4611163

[12] Kaiko GE Horvat JC Beagley KW Hansbro PM. Immunological decision-making: how does the immune system decide to mount a helper T-cell response? Immunology (2008) 123:326–38. 10.1111/j.1365-2567.2007.02719.x 17983439 PMC2433332

[13] Upchurch KC Boquin JR Yin W Xue Y Joo H Kane RR New TLR7 agonists with improved humoral and cellular immune responses. Immunol Lett (2015) 168:89–97. 10.1016/j.imlet.2015.09.007 26381186

[14] Liu X Li J Liu Y Ding J Tong Z Liu Y Calreticulin acts as an adjuvant to promote dendritic cell maturation and enhances antigen-specific cytotoxic T lymphocyte responses against non-small cell lung cancer cells. Cell Immunol (2016) 300:46–53. 10.1016/j.cellimm.2015.12.003 26702740

[15] Fu Y Wang T Xiu L Shi X Bian Z Zhang Y Levamisole promotes murine bone marrow derived dendritic cell activation and drives Th1 immune response in vitro and in vivo. Int Immunopharmacol (2015) 31:57–65. 10.1016/j.intimp.2015.12.015 26706452

[16] Kim SK Yun CH Han SH. Enhanced anti-cancer activity of human dendritic cells sensitized with gamma-irradiation-induced apoptotic colon cancer cells. Cancer Lett (2013) 335:278–88. 10.1016/j.canlet.2013.02.038 23485725

[17] Kim SK Yun CH Han SH. Dendritic cells differentiated from human umbilical cord blood-derived monocytes exhibit tolerogenic characteristics. Stem Cells and Dev (2015) 24:2796–807. 10.1089/scd.2014.0600 26203805

[18] Reshma CS Sruthi S Syama S Gayathri V Mohanan PV. Assessing the systemic toxicity in rabbits after sub acute exposure to ocular irritant chemicals. Toxicol Res (2015) 31:49–59. 10.5487/TR.2015.31.1.049 25874033 PMC4395655

[19] Khayrullina T Yen JH Jing H Ganea D. In vitro differentiation of dendritic cells in the presence of prostaglandin E2 alters the IL-12/IL-23 balance and promotes differentiation of Th17 cells. J Immunol (2008) 181:721–35. 10.4049/jimmunol.181.1.721 18566439 PMC2835359

[20] Staubach S Hanisch FG. Lipid rafts: signaling and sorting platforms of cells and their roles in cancer. Expert Rev Proteomics (2011) 8:263–77. 10.1586/EPR.11.2 21501018

[21] Christian AE Haynes MP Phillips MC Rothblat GH. Use of cyclodextrins for manipulating cellular cholesterol content. J Lipid Res (1997) 38:2264–72. 9392424

[22] Kamala T. Hock immunization: a humane alternative to mouse footpad injections. J Immunol Methods (2007) 328:204–14. 10.1016/j.jim.2007.08.004 17804011 PMC2464360

[23] Kusakabe T Ozasa K Kobari S Momota M Kishishita N Kobiyama K Intranasal hydroxypropyl-beta-cyclodextrin-adjuvanted influenza vaccine protects against sub-heterologous virus infection. Vaccine (2016) 34:3191–8. 10.1016/j.vaccine.2016.04.001 27160037

[24] Tanaka Y Yamada Y Ishitsuka Y Matsuo M Shiraishi K Wada K Efficacy of 2-hydroxypropyl-beta-cyclodextrin in Niemann-Pick disease type C model mice and its pharmacokinetic analysis in a patient with the disease. Biol Pharm Bull (2015) 38:844–51. 10.1248/bpb.b14-00726 26027824

[25] Senti G Iannaccone R Graf N Felder M Tay F Kundig T. A randomized, double-blind, placebo-controlled study to test the efficacy of topical 2-hydroxypropyl-beta-cyclodextrin in the prophylaxis of recurrent herpes labialis. Dermatology (2013) 226:247–52. 10.1159/000349991 23816977

[26] Flach TL Ng G Hari A Desrosiers MD Zhang P Ward SM Alum interaction with dendritic cell membrane lipids is essential for its adjuvanticity. Nat Med (2011) 17:479–87. 10.1038/nm.2306 21399646

[27] Ismaili J Rennesson J Aksoy E Vekemans J Vincart B Amraoui Z Monophosphoryl lipid A activates both human dendritic cells and T cells. J Immunol (2002) 168:926–32. 10.4049/jimmunol.168.2.926 11777991

[28] Bagley KC Abdelwahab SF Tuskan RG Fouts TR Lewis GK. Cholera toxin and heat-labile enterotoxin activate human monocyte-derived dendritic cells and dominantly inhibit cytokine production through a cyclic AMP-dependent pathway. Infect Immun (2002) 70:5533–9. 10.1128/IAI.70.10.5533-5539.2002 12228279 PMC128358

[29] Romera SA Hilgers LA Puntel M Zamorano PI Alcon VL Dus Santos MJ Adjuvant effects of sulfolipo-cyclodextrin in a squalane-in-water and water-in-mineral oil emulsions for BHV-1 vaccines in cattle. Vaccine (2000) 19:132–41. 10.1016/S0264-410X(00)00104-3 10924795

[30] Alpar HO Eyles JE Williamson ED Somavarapu S. Intranasal vaccination against plague, tetanus and diphtheria. Adv Drug Deliv Rev (2001) 51:173–201. 10.1016/S0169-409X(01)00166-1 11516788

[31] Didierlaurent AM Collignon C Bourguignon P Wouters S Fierens K Fochesato M Enhancement of adaptive immunity by the human vaccine adjuvant AS01 depends on activated dendritic cells. J Immunol (2014) 193:1920–30. 10.4049/jimmunol.1400948 25024381

[32] Bagley KC Abdelwahab SF Tuskan RG Lewis GK Cholera toxin indirectly activates human monocyte-derived dendritic cells in vitro through the production of soluble factors, including prostaglandin E(2) and nitric oxide. Clin Vaccine Immunol (2006) 13:106–15. 10.1128/CVI.13.1.106-115.2006 16426007 PMC1356627

[33] Bungener L Geeraedts F Ter Veer W Medema J Wilschut J Huckriede A. Alum boosts TH2-type antibody responses to whole-inactivated virus influenza vaccine in mice but does not confer superior protection. Vaccine (2008) 26:2350–9. 10.1016/j.vaccine.2008.02.063 18400340

[34] Wheeler AW Marshall JS Ulrich JT. A Th1-inducing adjuvant, MPL, enhances antibody profiles in experimental animals suggesting it has the potential to improve the efficacy of allergy vaccines. Int Arch Allergy Immunol (2001) 126:135–9. 10.1159/000049504 11729351

[35] Mattsson J Schon K Ekman L Fahlen-Yrlid L Yrlid U Lycke NY Cholera toxin adjuvant promotes a balanced Th1/Th2/Th17 response independently of IL-12 and IL-17 by acting on Gsalpha in CD11b(+) DCs. Mucosal Immunol (2015) 8:815–27. 10.1038/mi.2014.111 25425266

[36] Barral DC Cavallari M McCormick PJ Garg S Magee AI Bonifacino JS CD1a and MHC class I follow a similar endocytic recycling pathway. Traffic (2008) 9:1446–57. 10.1111/j.1600-0854.2008.00781.x 18564371 PMC3839101

[37] McCauliff LA Xu Z Storch J Sterol transfer between cyclodextrin and membranes: similar but not identical mechanism to NPC2-mediated cholesterol transfer. Biochemistry (2011) 50:7341–9. 10.1021/bi200574f 21740003 PMC4281486

[38] Atger VM Moya MD Stoudt GW Rodrigueza WV Phillips MC Rothblat GH. Cyclodextrins as catalysts for the removal of cholesterol from macrophage foam cells. J Clin Invest (1997) 99:773–80. 10.1172/Jci119223 9045882 PMC507862

[39] Triantafilou M Miyake K Golenbock DT Triantafilou K. Mediators of innate immune recognition of bacteria concentrate in lipid rafts and facilitate lipopolysaccharide-induced cell activation. J Cell Sci (2002) 115:2603–11. 12045230 10.1242/jcs.115.12.2603

[40] Yancey PG Rodrigueza WV Kilsdonk EP Stoudt GW Johnson WJ Phillips MC Cellular cholesterol efflux mediated by cyclodextrins. Demonstration of kinetic pools and mechanism of efflux. J Biol Chem (1996) 271:16026–34. 10.1074/jbc.271.27.16026 8663188

[41] Gherardi RK. [Lessons from macrophagic myofasciitis: towards definition of a vaccine adjuvant-related syndrome]. Rev Neurol (2003) 159:162–4. 12660567

[42] Asa PB Cao Y Garry RF. Antibodies to squalene in Gulf War syndrome. Exp Mol Pathol (2000) 68:55–64. 10.1006/exmp.1999.2295 10640454

[43] Bagavant H Nandula SR Kaplonek P Rybakowska PD Deshmukh US Alum, an aluminum-based adjuvant, induces Sjogren’s syndrome-like disorder in mice. Clin Exp Rheumatol (2014) 32:251–5. 24739520 PMC3990870

[44] Verstraeten T Descamps D David MP Zahaf T Hardt K Izurieta P Analysis of adverse events of potential autoimmune aetiology in a large integrated safety database of AS04 adjuvanted vaccines. Vaccine (2008) 26:6630–8. 10.1016/j.vaccine.2008.09.049 18845199

