# Supplementary material for: Induction of Dendritic Cell Maturation and Activation by a Potential Adjuvant, 2-Hydroxypropyl-β-Cyclodextrin
Source: Front Immunol. 2016 Oct 20;7:435. doi: 10.3389/fimmu.2016.00435 (PMC5071323; doi:10.3389/fimmu.2016.00435)
Supplement: Supplementary file 1 [file Data_Sheet_1.DOCX]

Supplementary Material

Induction of Dendritic Cell Maturation and Activation by a Potential Adjuvant, 2-Hydroxypropyl-β-cyclodextrin

**Sun Kyung Kim, Cheol-Heui Yun, Seung Hyun Han^*^**

*** Correspondence:** Seung Hyun Han: [shhan-mi@snu.ac.kr](mailto:shhan-mi@snu.ac.kr)

**Materials and methods**

**Cell viability analysis**

DCs (2.5 × 10^5^ cells/ml) were stimulated with either HP-β-CD (0, 0.1, 0.3, or 1 mg/ml) in the presence of GM-CSF (2.5 ng/ml) and IL-4 (5 ng/ml) for 24 h. The unstimulated or stimulated DCs (5 × 10^4^ cells) were suspended in 100 μl Annexin V-binding buffer (BD Biosciences) containing 1.25 μg/ml PI (Sigma-Aldrich) and 0.2 μg/ml FITC-conjugated Annexin V (BioLegend) and incubated for 10 min at room temperature. Cell viability was analyzed using flow cytometry with FACSCalibur and FlowJo software (TreeStar).

**Lymphocyte activation by** **HP-β-CD or HP-β-CD-sensitized DCs**

CD3^+^ T lymphocytes were isolated from CD14^+^ monocyte-depleted peripheral blood mononuclear cells by positive selection using CD3 magnetic beads (BD Biosciences). The isolated CD3^+^ T lymphocytes were labeled with 10 μM 5,6-carboxyfluorescein diacetate succinimidyl ester (CFSE) for 15 min at 37˚C and washed with PBS. The CFSE-labeled autologous CD3^+^ T lymphocytes (2.5 × 10^5^ cells/ml) were treated with HP-β-CD (0.3 or 1 mg/ml) for 4 days. In a separate experiment, immature DCs (2.5 × 10^5^ cells/ml) were treated with HP-β-CD (1 mg/ml) in the presence of GM-CSF (2.5 ng/ml) and IL-4 (5 ng/ml) for 16 h. After removal of the culture supernatant, the DCs (5 × 10^4^ cells) were co-cultured with the CFSE-labeled autologous CD3^+^ T lymphocytes (5 × 10^4^ cells) for 4 days. The proliferation and CD25 expression of the T lymphocytes were analyzed by flow cytometry and FlowJo software.

**Immunization with OVA plus HP-β-CD in mice**

Seven-week-old male C57BL/6 mice were purchased from Orient Bio (Sungnam, Korea) and maintained in a specific pathogen-free animal facility. All experiments using animals were conducted under the approval of the Institutional Animal Care and Use Committee of Seoul National University. Care and treatment of the animals were carried out in accordance with the approved guidelines. Mice were anesthetized by intraperitoneal injection of Avertin (2,2,2-tribromoethanol and 2-methyl-2-butanol) and administered with 20 μg OVA with or without 3 mg HP-β-CD through a hind footpad. The mice were maintained for 24 h or 7 days and sacrificed to obtain the draining lymph nodes and the blood, respectively.

**Measurement of antibody titers**

The blood samples were taken from the mice immunized with OVA with or without HP-β-CD on day seven by cardiac puncture and subjected to centrifugation at 11,460 ×*g*, 10 min. The sera were collected and stored at -80°C. Titers of OVA-specific antibodies (total IgG, IgG1, and IgG2a) in the sera were determined by ELISA using horseradish peroxidase (HRP)-conjugated anti-mouse total IgG, IgG1, and IgG2a antibodies (Southern Biotech). Briefly, 96-well microtiter plates were incubated with 10 μg/ml OVA in PBS overnight at 4°C. The plates were washed and incubated with 1% BSA in PBS for 1 h. The plates were washed and incubated with diluted serum for 2 h at room temperature. The plates were washed and incubated with HRP-conjugated anti-mouse total IgG, IgG1, or IgG2a antibody for 2 h. After the plates were washed, color reactions were initiated by the addition of TMB solution (BioLegend) and stopped with 2 N H_2_SO_4_. The optical density was measured at a wavelength of 450 nm. The antibody titers expressed as the reciprocal log_2_ were determined at the point giving an optical density at 450 nm higher than background (cutoff value: OD_450_=0.1).


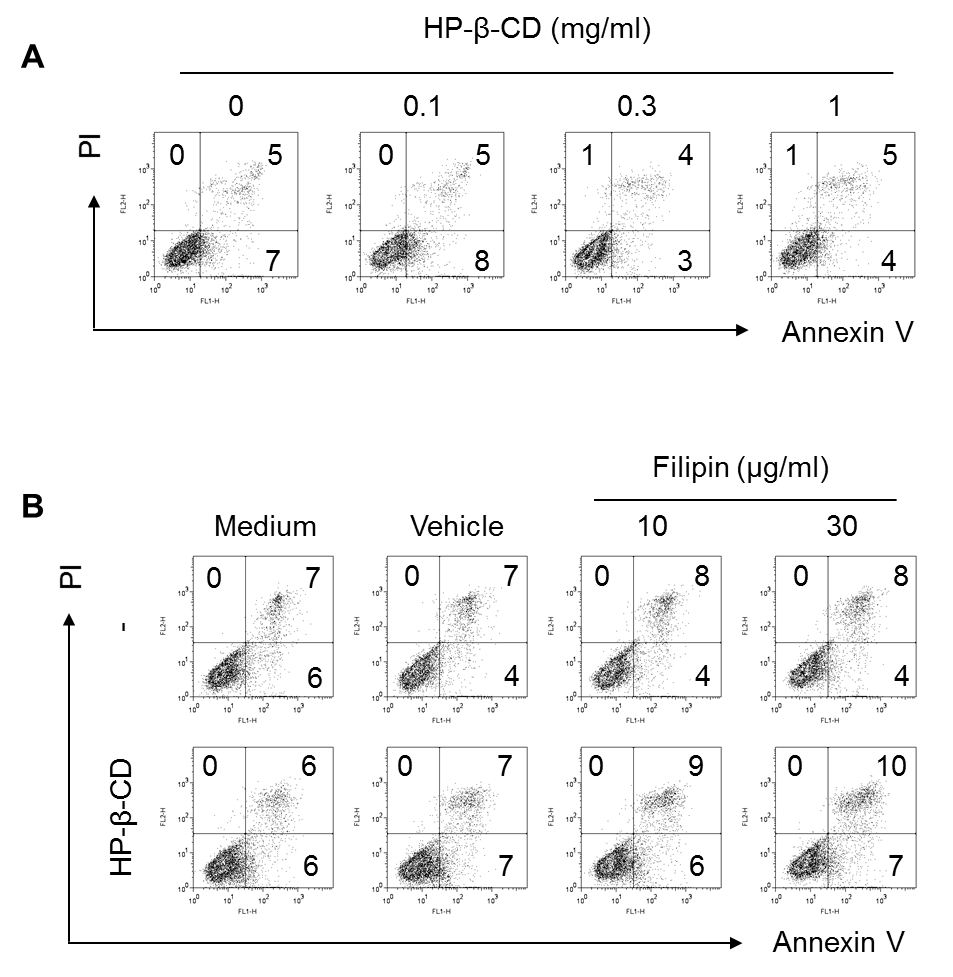


**Supplementary Figure 1.** **HP-β-CD is not cytotoxic to DCs.**

(A) Immature DCs (2.5 × 10^5^ cells/ml) were stimulated with HP-β-CD (0, 0.1, 0.3, or 1 mg/ml) in the presence of GM-CSF and IL-4 for 24 h. DCs stimulated with HP-β-CD were stained with Annexin V and PI and cell viability was determined by flow cytometry. (B) Immature DCs (2.5 × 10^5^ cells/ml) were pretreated with filipin (10 and 30 μg/ml) or methanol as a vehicle control for 1 h, followed by stimulation with HP-β-CD (1 mg/ml) for another 24 h. Cells were stained with Annexin V and PI, and then the cell viability was analyzed by flow cytometry.


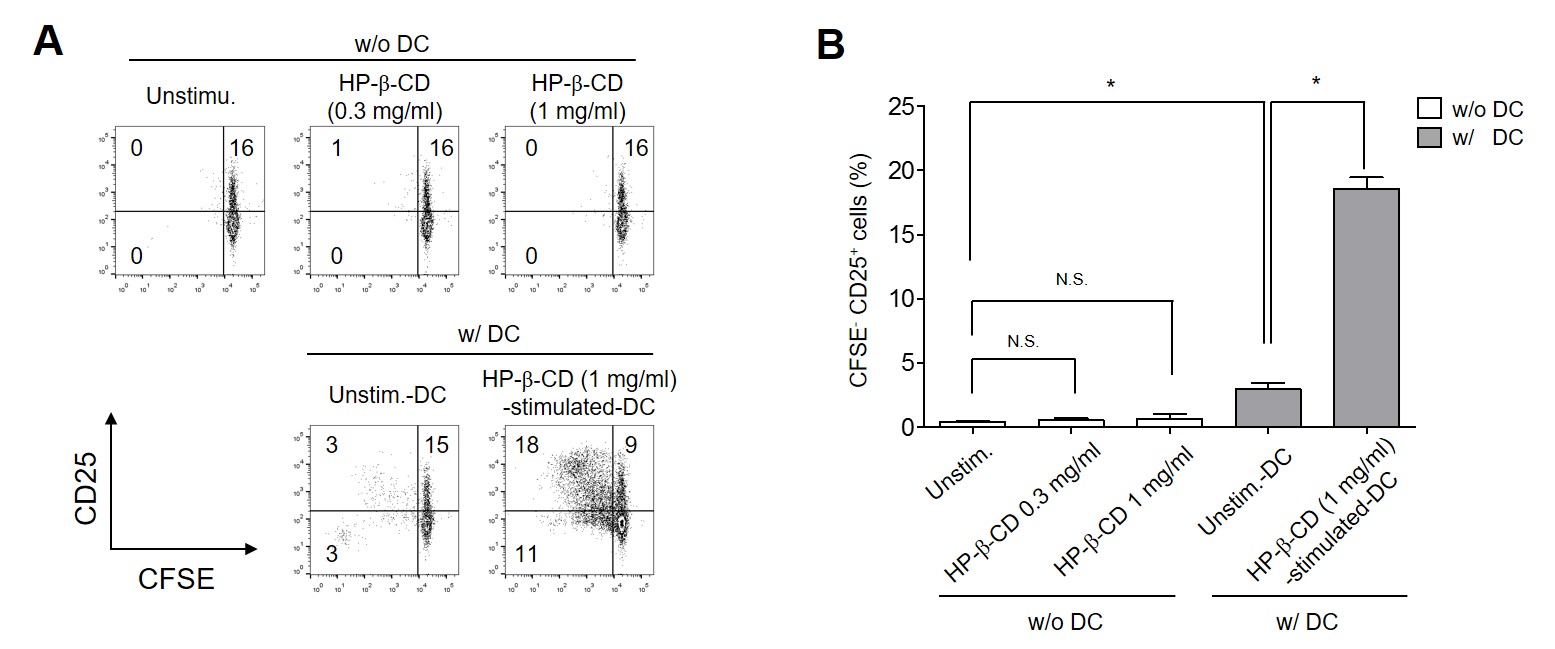


**Supplementary Figure 2.** **HP-β-CD-sensitized DCs, but not HP-β-CD, activate T lymphocytes.**

Human monocyte-derived DCs (2.5 × 10^5^ cells/ml) were stimulated with HP-β-CD (0 or 1 mg/ml) for 16 h. Then, the DCs (5 × 10^4^ cells) were co-cultured with CFSE-labeled autologous CD3^+^ T cells (5 × 10^4^ cells) for 4 days. In a separate experiment, the CD3^+^ T lymphocytes (2.5 × 10^5^ cells/ml) were stimulated with HP-β-CD (0.3 or 1 mg/ml) in the absence of DCs for 4 days. (A) The proliferation and CD25 expression of T lymphocyte were analyzed by flow cytometry. The numbers on the histograms indicate the percentages of T lymphocytes in each quadrant. (B) The bar graph displays the average of triplicate measurements for the proliferation of CD25^+^ T lymphocytes. Statistical significance was analyzed by Student’s *t*-test. N.S., not significant; *, *P* < 0.05. Unstim.-DC indicates T lymphocytes cocultured with unstimulated DCs.


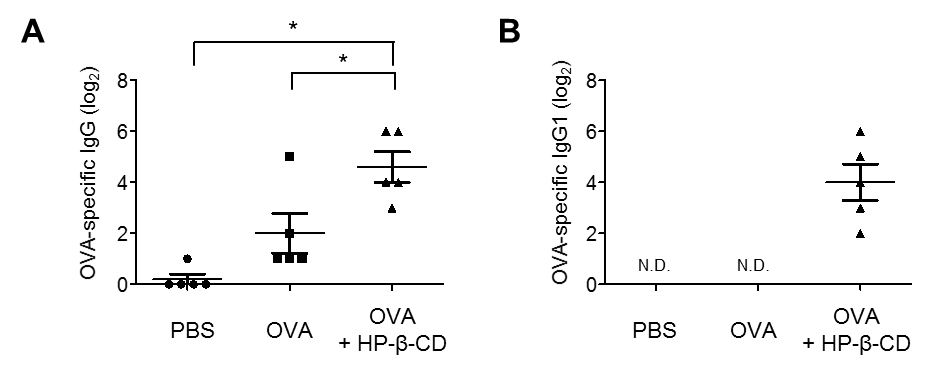


**Supplementary Figure 3.** **HP-β-CD potentiates humoral immune responses to coadministered OVA antigens.**

C57BL/6 mice were immunized with 20 μg OVA in the presence or absence of 3 mg HP-β-CD through a hind footpad. Seven days after the immunization, titers of OVA-specific (A) total IgG or (B) IgG1 were measured in the blood by ELISA. *, *P* < 0.05. The result shown is a representative of four similar experiments.
